# Bioprosthetic Aortic Valve on the Move

**DOI:** 10.1016/j.case.2022.07.002

**Published:** 2022-09-13

**Authors:** Ioannis Milioglou, Carl Guillombardo, Farshad Forouzandeh

**Affiliations:** Harrington Heart and Vascular Institute, University Hospitals, Cleveland, Ohio

**Keywords:** Bioprosthetic valve, Acute aortic regurgitation, Valvular heart disease, Cardiogenic shock

## Abstract

• Acute bioprosthetic aortic valve regurgitation can present with cardiogenic shock.
• Echocardiography is critical in the assessment of bioprosthetic aortic valve function.
• In up to 6% of patients with infectious endocarditis, no microbiologic agent is identified.
• Surgery is the mainstay of treatment for prosthetic acute aortic regurgitation.

## Graphical abstract

**Figure:**
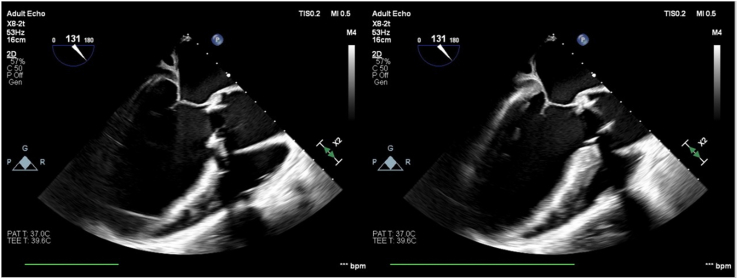

## Introduction

Surgery is the mainstay of treatment for patients with acute aortic regurgitation (AR).^1^^,^^2^ Bioprosthetic valves (BVs) demonstrate an excellent profile in terms of hemodynamics and seem to be a good fit for patients with high risk of bleeding. Nonetheless, the durability of BVs is limited compared to mechanical valves; inevitably, BV degeneration may lead to significant complications requiring major surgical reintervention.^3^, ^4^, ^5^

## Case Presentation

A 44-year-old male patient with no medical history presented with progressive dyspnea on exertion for 1 month (Table 1). The primary care physician noticed a new systolic murmur on exam, and echocardiography was notable for severe AR, reduced ejection fraction of 40%, and end-diastolic left ventricular diameter of 61 mm. Coronary angiography was normal. A computed tomography with angiography of the chest showed normal aorta throughout its course. Autoimmune panel, syphilis titers, and blood cultures were negative. Intraoperatively, an underdeveloped and low implanted commissure (3-4 mm compared to the other commissures) between the non- and right coronary cusps was noted (Videos 1 and 2). Histopathology examination of the aortic valve leaflets revealed mild fibrotic changes and nonspecific myxoid degeneration indicative of myxomatous degeneration. A 25-mm Carpentier-Edwards Perimount Magna Ease BV was placed without postplacement valvular AR or perivalvular leak (PVL). The patient returned to the previous normal functional status.

**Table 1:** Timeline

Time	Event
T –6 months	Initial presentation at primary care physician
T –4 months	First BV implantation
T –24 hours	Current presentation at peripheral hospital
T 0	Admission to cardiologic intensive care unit
T +12 hours	Second operating room, BV replacement
T +16 days	Discharge on intravenous antibiotics

**Figure 1:**
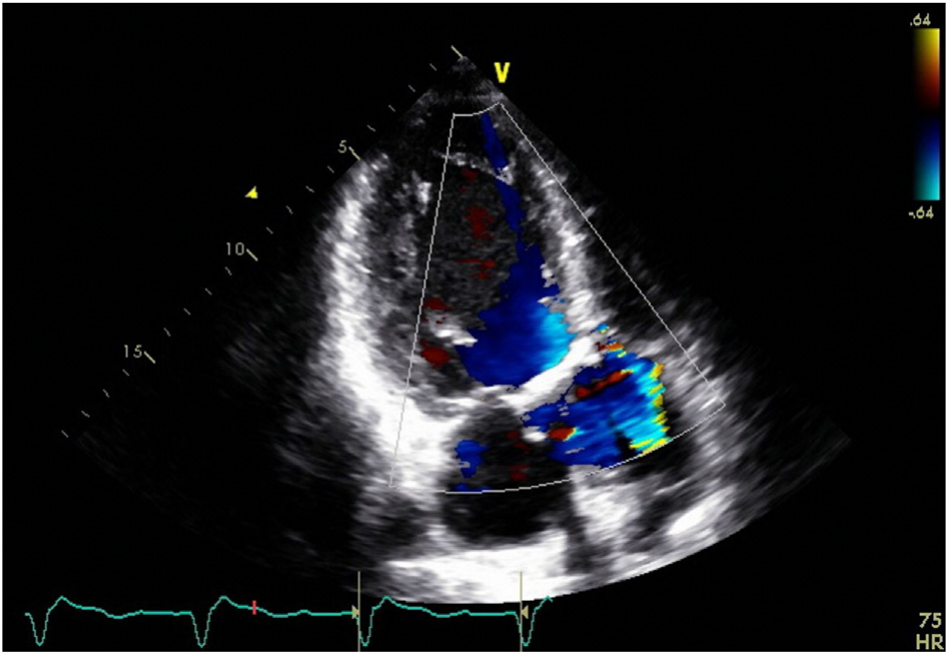
Transthoracic 3-chamber view with color Doppler across the bioprosthetic aortic valve during end systole. Accelerated anterior paravalvular jet.

**Figure 2:**
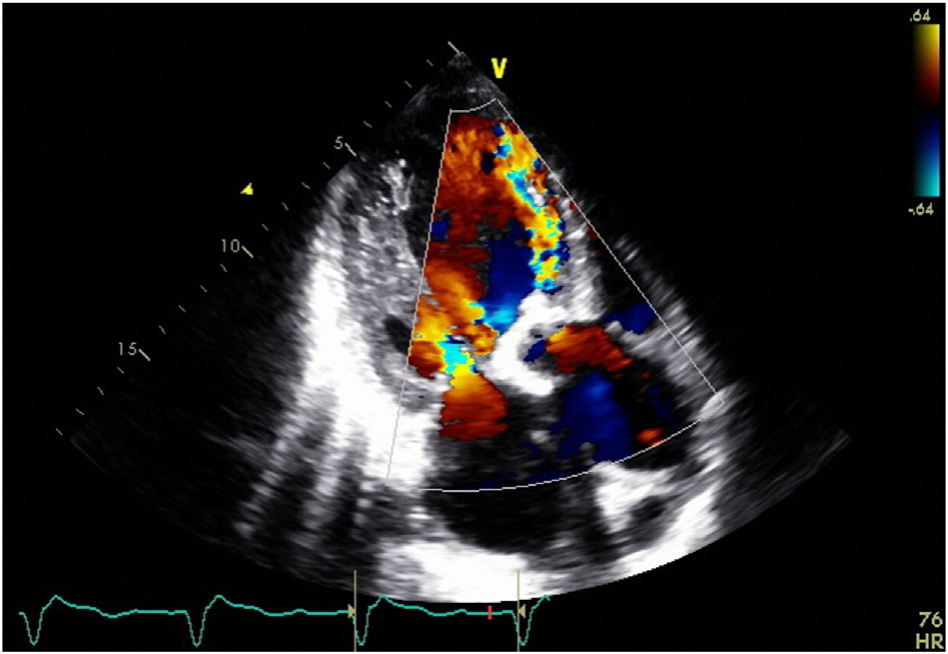
Transthoracic 3-chamber view with color Doppler across the bioprosthetic aortic valve during end diastole. Eccentric regurgitation jet as well as diastolic rocking of the valve in the left ventricle compared to end-systolic valve position.

**Figure 3:**
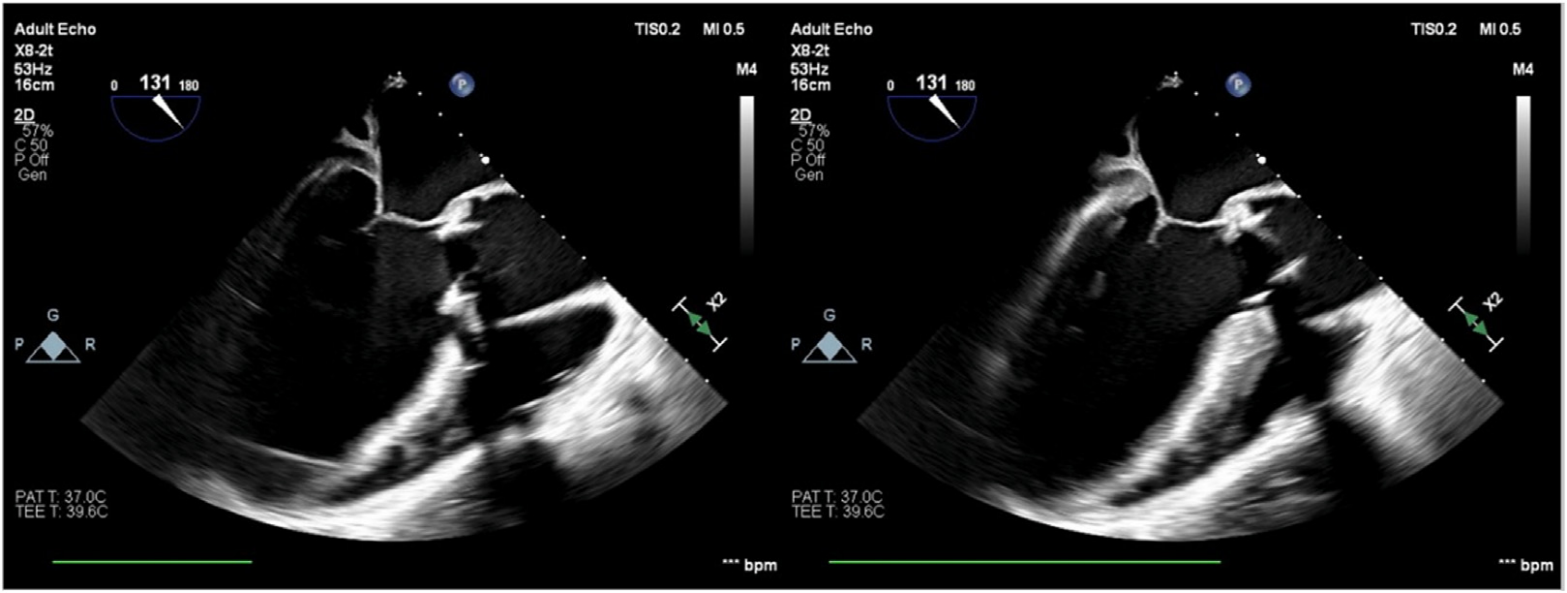
Intraoperative transesophageal echocardiogram, midesophageal long-axis view without color Doppler at end diastole *(left)* and end systole *(right)*, omniplane angle at 131°. Bioprosthetic aortic valve with anterior dehiscence as noted by the superiorly displaced anterior part of the valve during systole compared to end diastole.

Six months after the operation, the patient presented again complaining of acute shortness of breath for 3 days. The patient denied fevers, chills, recent travels, recreational drug use, or recent dental work. Initial blood pressure was 140/50 mm Hg. A bedside right heart catheterization was also performed (right atrial pressure, 19 mm Hg; right ventricular systolic/diastolic and mean pressure, 50/30/35 mm Hg; wedge pressure, 35 mm Hg; cardiac output/cardiac index, 2.2 L/min/1.1 L/min/m^2^; systemic vascular resistance, 901 d/cm). Initial lactate was 13 mg/dL, and creatinine was 1.7 mg/dL from a baseline of 0.9 mg/dL. A new echocardiogram showed severely decreased ejection fraction of 25% to 30%, severe PVL, and an abnormal bioprosthetic aortic valve with dehiscence prolapsing into the left ventricle (Videos 3 and 4, Figures 1 and 2). The patient was urgently taken to the operating room in the setting of worsening multiorgan dysfunction syndrome and negative blood cultures for 36 hours. Intraoperative findings confirmed PVL due to prosthetic valve dehiscence (Video 5, Figure 3). A 29-mm INSPIRIS valve was placed. No AR or PVL were noted on postop transesophageal echocardiography (Video 6). Histopathologic examination this time showed neutrophilic inflammation of the perivalvular tissue and fibrinous material, raising suspicion for an infectious process. Blood cultures, intraoperative tissue cultures, and 16S RNA assays were all negative for bacterial growth. The patient was discharged on postop day 16 on cefepime, vancomycin, rifampin, and gentamycin for 6 weeks for a presumed diagnosis of culture-negative endocarditis.

## Discussion

We present the case of an acutely failing BV in the setting of culture-negative endocarditis requiring emergent redo surgical valve replacement. Over the last 2 decades, the use of BV has been increasing steadily.^6^ Therefore, cardiologists will manage more patients with failed BV in the coming years. There are 4 main modes of BV failure: structural (calcifications, leaflet tear, dehiscence, wear, fracture, embolization), nonstructural (pannus, suture entrapment, PVL, disproportion), thrombosis, and endocarditis (abscess, dehiscence, vegetation, cusp damage, fistula).^7^ Bioprosthetic valve failure etiologies can also be classified as early and late based on the timing from operation. Early-failure etiologies are mostly related to technical challenges during surgery or early endocarditis. Intraoperative calcium debridement, repeat surgery, and aortic annulus reconstruction are associated with higher rates of early PVL, most of which are mild and not clinically relevant. Last, early acute endocarditis occurs in less than 1% of the patients. Late BV failure etiologies include pannus formation, which leads to valve stenosis and most frequently valve degeneration, which can lead to either stenosis or regurgitation.^1^ There are several factors affecting BV durability such as valve position and patient age. Aortic valve bioprostheses have better durability compared with their mitral counterparts. Finally, BVs fail earlier in younger patient populations compared with older patients.^8^

Transthoracic echocardiography (TTE) is the first imaging modality that is employed to assess the function of a prosthetic valve. When it comes to aortic BV failure, TTE is useful to identify the presence of either AR or PVL given the lack of acoustic shadowing that is often seen in mitral BV evaluation. The key views on TTE when assessing for AR include parasternal long-axis and short-axis views, the 5-chamber view, and the apical long-axis views. Quantification of severe AR is based on qualitative and quantitative Doppler parameters including large central regurgitant jet, decreased and steep pressure half-time (<200 m/sec), holodiastolic diastolic flow reversal in the descending aorta, regurgitant volume of >60 mL/beat, and regurgitant fraction >50%.^9^

Infective endocarditis usually occurs within 2 years of valve implant, and up to 6% of patients with BVs can be affected. *Staphylococcus epidermidis* and *Staphylococcus aureus*, streptococci, Gram-negative bacilli, and fungi are the most commonly identified organisms. Mononuclear cells are also reported on pathologic specimens of valves when explanted.^10^ In 6% of cases with infective endocarditis, no organisms are isolated. Culture-negative endocarditis is defined as endocarditis with at least 3 blood culture samples that are negative after 7 days of incubation and subculturing. The main reasons for culture-negative IE are iatrogenic (poor microbiological techniques, previous antibiotic administration) or highly fastidious organisms or fungi. In these patients, polymerase chain reaction testing of the pathologic specimens can lead to an infectious etiology; its sensitivity in certain setting reaches 100%.^11^

Transthoracic echocardiography may demonstrate BV regurgitation or stenosis in IE. Abscess formation on the suturing ring destroys the annular tissues and the valve cusp, resulting in valvular regurgitation. The reported sensitivity and specificity of TTE in detecting abscesses are 28% and 98%, respectively, compared with, 87% and 95% on transesophageal echocardiography.^12^ Bioprosthetic valve infective endocarditis can also result in cusp perforations leading to regurgitation. Finally, large vegetations may obstruct cusp opening and closure, resulting in a stenotic valve.^7^

Even though redo surgery is the mainstay of treatment for BV failure, it is associated with a greater than 2-fold increase in perioperative mortality compared to the primary aortic valve replacement. Thirty-day mortality of redo aortic prosthetic surgery is reported to be as high as 5.4%. On the other hand, a transcatheter valve-in-valve approach can be a promising alternative to redo surgery for high-risk patients but only in patients without active endocarditis and dehiscence. A recent meta-analysis comparing valve-in-valve to redo aortic valve surgery reported similar 30-day and 1-year mortality rates (4.4 vs 5.7%, *P* = .83; hazard ratio, 0.93; *P* = .51). Nonetheless, redo surgery was associated with lower rates of postoperative PVL and patient-prostheses mismatches.^13^

## Conclusion

Bioprosthetic valve failure can manifest with a wide range of clinical presentations depending on its severity and the underlying mechanism. Transthoracic echocardiography is a useful tool to assess both the severity and etiology of BV failure, which guides further diagnostic and therapeutic planning.

## Supplementary Data

Video 1 Transesophageal echocardiogram, midesophageal off-axis long-axis view without color Doppler, omniplane angle at 118°. Normal appearing left ventricular outflow tract as well as noncoronary *(upper)* and right coronary cusps.

Video 2 Transesophageal echocardiogram, midesophageal off-axis long-axis view with color Doppler, omniplane angle at 118°. Eccentric anteriorly directed aortic regurgitation jet.

Video 3 Transthoracic 3-chamber view with color Doppler across the bioprosthetic aortic valve. Eccentric regurgitation jet as well as diastolic rocking of the valve in the left ventricle.

Video 4 Transthoracic short-axis view zoomed-in aortic valve with color Doppler shows dehiscence of the BV from the aortic root. Regurgitant jet neck width at the level of sewing ring ∼60% of the area consistent with severe eccentric aortic regurgitation.

Video 5 Intraoperative transesophageal echocardiogram, midesophageal long-axis view without color Doppler, omniplane angle at 131°, Dilated left ventricle, bioprosthetic aortic valve with anterior dehiscence with rocking motion with each cardiac cycle.

Video 6 Intraoperative transesophageal echocardiogram, midesophageal long-axis view with color Doppler, omniplane angle at 148°. Postbypass and postreplacement of aortic BV with a new INSPIRIS 29 mm, without signs of valvular or paravalvular leak.
